# Altered GABAergic Homeostasis in the Striatum of Dopamine Transporter Knockout Rats

**DOI:** 10.2174/011570159X370747250404060428

**Published:** 2025-04-18

**Authors:** Giorgia Targa, Beatrice Rizzi, Francesca Mottarlini, Raul R. Gainetdinov, Damiana Leo, Fabio Fumagalli, Lucia Caffino

**Affiliations:** 1 Department of Pharmacological and Biomolecular Sciences ‘Rodolfo Paoletti’, Università degli Studi di Milano, Via Balzaretti 9, 20133, Milan, Italy;; 2 Center for Neuroscience, University of Camerino, Camerino, Italy;; 3 Institute of Translational Biomedicine, St. Petersburg State University, 7/9 Universitetskaya Emb., 199034 St. Petersburg, Russia;; 4 St. Petersburg University Hospital, St. Petersburg State University, Fontanka River Emb. 154, 190121 St. Petersburg, Russia;; 5 Department of Neurosciences, University of Mons, 6 Avenue du Champ de Mars, 7000, Mons, Belgium

**Keywords:** Dopamine transporter, striatum, GABA, GAD67, GABA-A receptors, dopamine homeostasis

## Abstract

**Background:**

It is now widely established that dopamine, despite its nature as a slow-acting biogenic monoamine, modulates fast neurotransmitters such as GABA. However, the mechanism through which this occurs still needs to be fully elucidated. The dopamine transporter (DAT) is the primary regulator of dopamine homeostasis, controlling extracellular levels of dopamine as well as its storage in vesicles.

**Methods:**

Here, we took advantage of the availability of dopamine transporter knockout (DAT^-/-^) rats, which provide a unique opportunity to investigate the response of the GABAergic system under hyperactivity of the dopaminergic system, a condition found in different disorders of the Central Nervous System. The expression levels of GABAergic markers have been evaluated by means of western blot in the whole homogenate, cytosolic fraction, and post-synaptic density of the striatum of male DAT^-/-^ rats.

**Results:**

We found a widespread down-regulation of GABAergic markers in the striatum of DAT^-/-^ rats. Our data show that DA overactivity critically reorganizes the striatal GABAergic synapse in a way that GABA neurotransmission appears to be toned down. Such changes are equally distributed among proteins regulating GABA synthesis (GAD67), release (vGAT) and reuptake (GAT1, GAT3). It also involves the main subunits of GABA receptors (GABA-A α1, α2, β1; GABA-B R1), their anchoring proteins (Gephyrin) and adhesion molecules (Neuroligin-2).

**Conclusion:**

Taken together, such changes paint a picture showing a compromised integrity of the striatal GABAergic system under conditions of functional hyperdopaminergia, which may be of interest for several disorders of the central nervous system.

## 1 INTRODUCTION

The dopaminergic neurotransmission is tightly regulated by the dopamine transporter (DAT), also known as SLC6A3, which belongs to a family of plasma membrane transporters of solute carrier family 6 (SLC6). Its main task is to reuptake released dopamine back from extracellular space into presynaptic terminals, thus maintaining physiological levels of dopamine in the synaptic cleft and re-filling dopaminergic vesicles for subsequent release. From a pharmacological point of view, DAT is also the major target for psychostimulants, such as cocaine. We have recently created dopamine transporter knockout rats (DAT^-/-^ rats) [1], which resemble the neurochemical, behavioral, and pharmacological features of DAT^-/-^ mice [2, 3].

In brief, DAT^-/-^ rats are hyperactive due to the high extracellular level of striatal dopamine. Furthermore, they exhibit dopamine depletion in intraneuronal storage vesicles, highlighting the critical role of DAT in their replenishment. Such dramatic dysregulation of dopamine homeostasis results in striking behavioral phenotypes involving cognitive and locomotor deficits [1] as well as in dysregulation of the glutamatergic system in the medial prefrontal cortex [4] and striatum [5]. Evidence exists showing that dopamine terminals form synapses with medium-spiny GABAergic neurons in the striatum [6, 7], suggesting that dopamine receptors regulate the activity of striatal GABAergic currents [8]. The opposite is also true, with GABA controlling the activity of dopaminergic neurons in the brain [9, 10]. Moreover, it was also suggested that the decreased striatal volume observed in DAT^-/-^ rodents, which might be partly responsible for the hyperactive behavior, could be explained by a loss of GABAergic interneurons, further corroborating the tight relationship between the two systems [11, 12]. The large majority of neurons in the striatum are projection neurons, the so-called medium spiny neurons (MSN), which account for the vast majority of total striatal cells, whose activity and synchronization are modulated by different types of GABAergic interneurons [13]. Such interneurons are densely expressed in the rodent striatum, accounting for the remaining percentage of striatal neurons and powerfully controlling striatal excitability by modulating and/or controlling the firing of MSN and maintaining a correct balance between the excitatory/inhibitory neurotransmission in the brain [14]. The stimulation of dopaminergic receptors with agonists can promote the activity of such striatal interneurons, whereas antagonists depress their firing frequency [15, 16]; moreover, dopamine itself may also influence interneuron activity *via* presynaptic D_2_ receptors [17].

GABA exerts a fast and strong inhibition of the striatum through stimulation of GABA-A receptors on striatal MSNs [18]. The GABA-A receptor is formed by multiple, distinct subunits, which differ in terms of function and brain region specificity [19] and form a ligand-gated ion pore that is permeable to Cl^-^ [20]. The presence of specific GABA-A receptor subtypes defines specific neuronal and functional networks; for instance, α1 GABA-A receptors appear to mediate sedation and cognitive impairments, whereas α2 GABA-A receptors modulate anxiolysis [21]. Instead, the slower action of GABA is mediated by the GABA-B receptor, a G protein-coupled receptor. Nevertheless, inhibitory neurotransmission primarily depends on GABA acting on GABA-A receptors, whose expression, assembly, and resulting functional changes are crucial for regulating the strength of GABAergic synapses [22-24]. Another critical player involved in the GABAergic synapse is neuroligin 2 (NL-2), a structural constituent of the inhibitory synapse, pivotal for GABAergic synapse formation and function. NL-2 is an adhesion molecule constitutively present at the GABAergic postsynaptic terminal, with the role of triggering the recruitment of inhibitory neurotransmitter receptors as well as the scaffolding molecule gephyrin [25, 26], whose function is to cluster GABA-A receptors and stabilize the inhibitory synapses [27].

DAT^-/-^ rats provide the unique and unprecedented possibility to study how GABAergic striatal homeostasis is modulated by dopamine, indeed representing an important step in understanding the physiology of the basal ganglia. Accordingly, we have investigated the different components of the presynaptic GABAergic transmission, focusing on the GABA release- and reuptake-associated proteins (vGAT, GAD65, GAD67, GAT1 and GAT3), the major subunits of the GABA-A receptor (α1 GABA-A receptors, α2 GABA-A receptors and β_1_ GABA-A receptors) as well as GABA-B R1 and its scaffolding protein gephyrin, the major GABAergic adhesion molecule neuroligin 2 (NL-2), the main calcium-binding proteins expressed in the GABAergic interneurons, *i.e*., parvalbumin and calbindin, in an attempt to draw a comprehensive picture of the neuroadaptations taking place under a situation of dopaminergic hyperactivity. We have also investigated the expression of molecules that, although not GABAergic in nature, nevertheless influence the GABAergic synapse, such as the peroxisome proliferator-activated receptor-gamma coactivator (PGC)-1α, a transcription factor localized into the nucleus of GABAergic neurons where it regulates gene expression [28] or reelin, a secreted glycoprotein synthesized by GABAergic neurons [29], which modulates synaptic plasticity both at presynaptic and postsynaptic sites [30].

Together, the data herein collected indicate an overall reduction in the GABAergic tone under conditions of striatal hyperdopaminergia.

## 2 MATERIALS AND METHODS

### 2.1 Animals

ZFN design, construction, *in vitro* validation, microinjection, and founder selection were performed as previously described [31, 32]. The ZFN Target site was: CTCATCAACCCGCCACAGAcaccaGTGGAGGCTCAAGAG in the Exon 2 of Slc6a3 gene (NCBI Gene ID: 24898; Genomic NCBI Ref Seq: NC_005100.3; mRNA NCBI Ref Seq: NM_012694.2). Sage Labs generated the knockout rat lines (DAT^-/-^) in the outbred Wistar Han background. 3 to 4 adult rats of DAT^-/-^ or DAT^+/+^ were maintained with free access to tap water and standard pellet food at 22°C and on a 12/12 h light/dark cycle (lights on 0700-1900 h). Genotyping was performed by PCR followed by enzymatic digestion with BtsI MutI (New England Biolabs, Milan, Italy) as previously described [1]. 3-month-old male DAT^-/-^ (250-275 g) and DAT^+/+^ (325-350 g) rats have been used.

### 2.2 Preparation of Protein Extract and Western Blot Assays

**Fig. (1):**
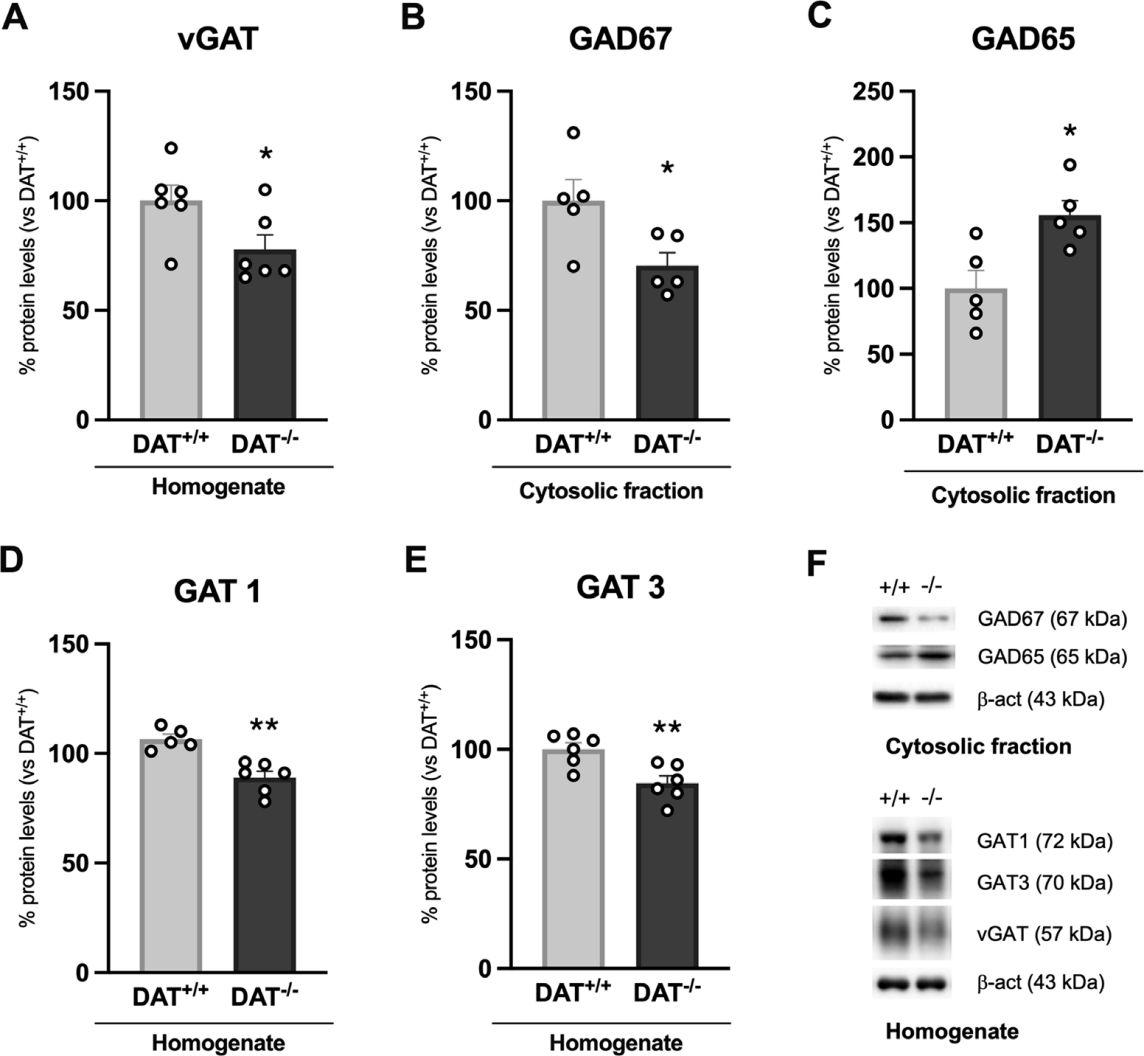
Protein expression levels of presynaptic GABAergic determinants and proteins involved in GABA reuptake. vGAT (**A**), GAT1 (**D**) and GAT3 (**E**) were investigated in the whole homogenate. GAD67 (**B**) and GAD65 (**C**) were measured in the cytosolic fraction. In (**F**), representative immunoblots for vGAT, GAD67, GAD65, GAT1, GAT3 and β-actin are shown. Data are expressed in scatter plot bar graphs as percentages of DAT^+/+^ rats and are expressed as mean ± standard error. Unpaired Student’s t-test *p<0.05, **p<0.01 *vs.* DAT^+/+^ rats.

**Fig. (2):**
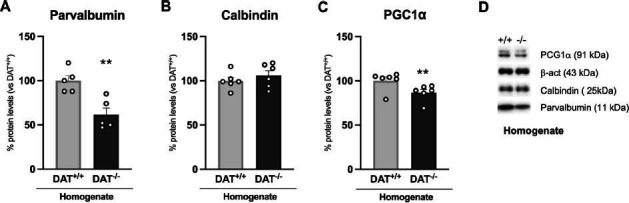
Protein expression levels of proteins involved in the proper functioning of the GABAergic neurotransmission. Parvalbumin (**A**), Calbindin (**B**) and PGC1α (**C**) were investigated in the whole homogenate. In (**D**) representative immunoblots for parvalbumin, calbindin, PGC1α and β-actin are shown. Data are expressed in scatter plot bar graphs as percentages of DAT^+/+^ rats and are expressed as mean ± standard error. Unpaired Student’s t-test or Mann-Whitney test **p<0.01 *vs.* DAT^+/+^ rats.

**Fig. (3):**
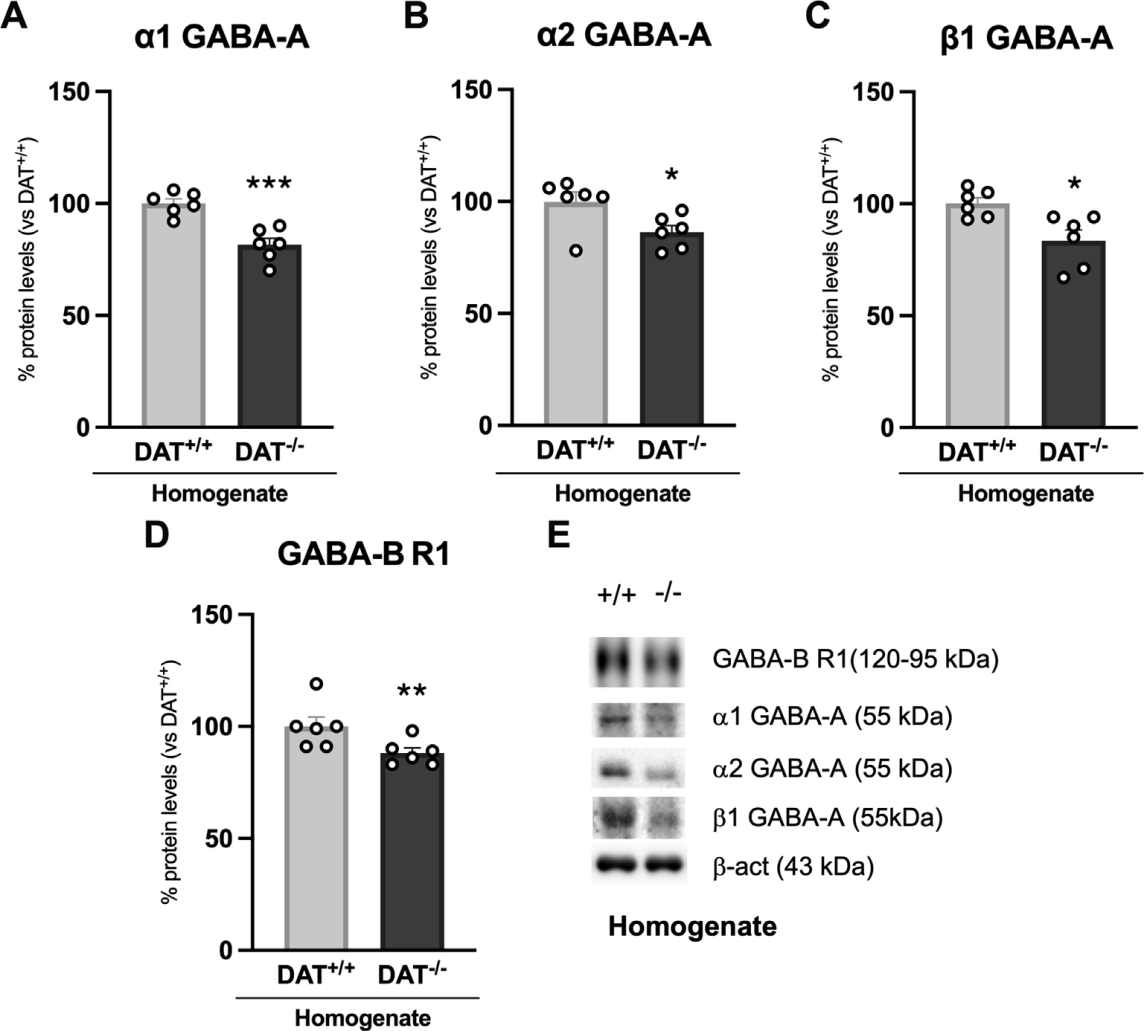
Protein expression levels of GABA-A receptor subunits and GABA-B receptor 1. α1 GABA-A (**A**), α2 GABA-A (**B**), β1 GABA-A (**C**) and GABA-B R1 (**D**) were investigated in the whole homogenate. In (**E**) representative immunoblots for α1 GABA-A, α2 GABA-A, β1 GABA-A, GABA-B R1 and β-actin are shown. Data are expressed in scatter plot bar graphs as percentages of DAT^+/+^ rats and are expressed as mean ± standard error. Unpaired Student’s t-test or Mann-Whitney test *p<0.05, **p<0.01, ***p<0.001 *vs*. DAT^+/+^ rats.

**Fig. (4):**
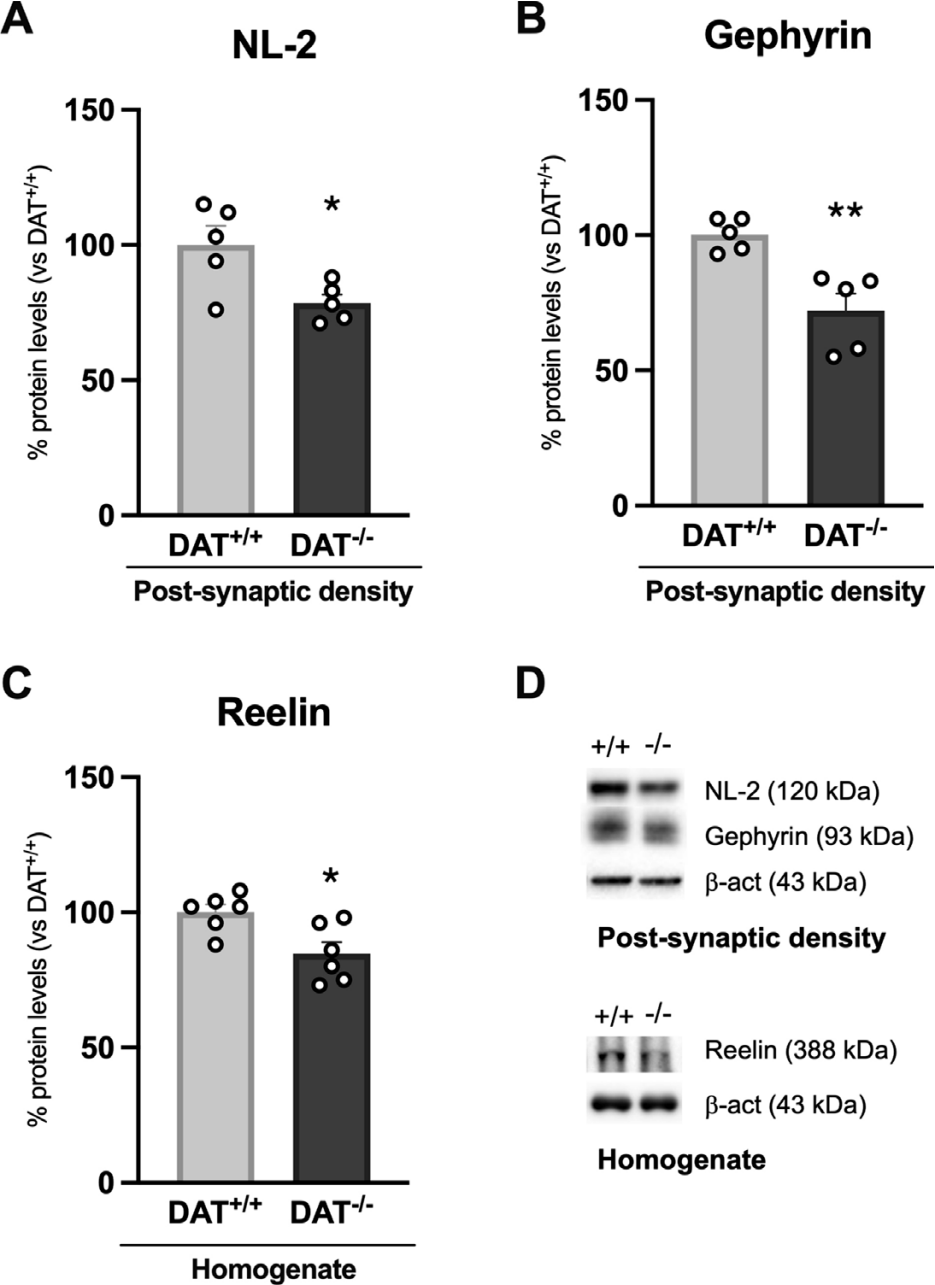
Protein expression levels of GABAergic scaffold and structural determinants. NL-2 (**A**) and gephyrin (**B**) were measured in the post-synaptic density. Reelin (**C**) was investigated in the whole homogenate. In (**D**) representative immunoblots for NL-2, gephyrin, reelin and β-actin are shown. Data are expressed in scatter plot bar graphs as percentages of DAT^+/+^ rats and are expressed as mean ± standard error. Unpaired Student’s t-test *p<0.05, **p<0.01 *vs*. DAT^+/+^ rats.

Proteins in the whole homogenate, post-synaptic, and cytosolic fraction of the striatum were extracted and analyzed as previously described with minor modifications [33]. Briefly, striata from adult male rats DAT^+/+^ (n = 6) and DAT^-/-^ (n = 6) were homogenized in a Teflon-glass potter in cold 0.32 M sucrose buffer pH 7.4 containing 1 mM HEPES, 1 mM MgCl_2_, 1 mM NaHCO_3,_ and 0.1 mM PMSF, in the presence of commercial cocktails of protease (Roche, Monza, Italy) and phosphatase inhibitors (Sigma-Aldrich, Milan, Italy). An aliquot of each homogenate was sonicated and stored at -20°C. The remaining homogenate was centrifuged at 800 g for 5 min; the resulting pellet (P1), corresponding to the nuclear fraction, was resuspended in a buffer (HEPES, 20 mM; dithiothreitol, 0.1 mM; EGTA, 0.1 mM) with protease and phosphatase inhibitors. Part of the resulting supernatant, corresponding to the cytosolic fraction, was stored for further analysis, whereas another aliquot of the supernatant was then centrifuged at 13000 g for 15 min, obtaining a pellet. This pellet was resuspended in a buffer containing 75 mM KCl and 1% Triton X-100 and centrifuged at 100,000 g for 1 h. The resulting pellet, referred to as PSD or Triton X-100 insoluble fraction (TIF, post-synaptic density), was homogenized in a glass-glass potter in 20 mM HEPES, protease and phosphatase inhibitors and stored at -20°C in the presence of glycerol 30%. The amount of proteins has been calculated in the homogenate, cytosolic, and TIF fractions by means of the Bradford Protein Assay procedure (Bio-Rad, Milan, Italy), using bovine serum albumin as the calibration standard [34]. Equal quantities of proteins from the homogenate (10 μg), TIF fraction (8 μg), and cytosolic fraction (10 μg) were separated on a sodium dodecyl sulfate 8% polyacrylamide gel under reducing conditions and then electrophoretically transferred onto nitrocellulose membranes (GE Healthcare, Milan, Italy). The entire nitrocellulose blot was separated into strips based on the molecular weight at which protein bands are anticipated to be detected, as indicated by their specific molecular weight and the datasheet of the antibody [35]. Blots were blocked for 1 h at room temperature with I-Block solution (Life Technologies Italia, Italy) in TBS 0.1% Tween-20 buffer and incubated with antibodies against the proteins of interest (Figs. **1**-**4**). The conditions of the primary antibodies were the following: anti parvalbumin (1:1000, Abcam, cod. ab11427, RRID: AB_298032), anti-vGAT (1:2000, Genetex, cod. GTX101908, RRID: AB_10619521), anti GAD65 (1:1000, Millipore, cod. AB1511, RRID: AB_90715), anti GAD67 (1:1000, Abcam, cod. ab26116, RRID: AB_448990), anti GAT1 (1:2000, Abcam, cod. ab426, RRID: AB_2189971), anti GAT3 (1:2000, Abcam, cod. ab122430, RRID: AB_11127719), anti α1 GABA-A receptors (1:500, StressMarq Biosciences, cod. SMC-346D-STR, RRID: AB_2700770), anti α2 GABA-A receptors (1:500, StressMarq Biosciences, cod. SMC-486D-A594, RRID: AB_2702463), anti β_1_ GABA-A receptors (1:500, Novus Biological, cod. NBP1-48319, RRID: AB_11033197), anti GABA-B R1 (1:1000, Cell Signaling Technology, cod. 3835, RRID: AB_2278774), anti NL-2 (1:1000, Synaptic System, cod. 129511, RRID: AB_2619813), anti Gephyrin (1:1000, Synaptic System, cod. 147111, RRID: AB_2619837), anti Reelin (1:1000, Abcam, cod. ab78540, RRID: AB_1603148), anti Calbindin (1:1000, Cell Signaling Technology, cod. 13176, RRID: AB_2687400), and anti β-actin (1:5000, Sigma-Aldrich, cod. A5441, RRID: AB_476744). Results were standardized using β-actin as the control protein, which was observed at 43 kDa. Immunocomplexes were visualized by chemiluminescence using the Chemidoc MP Imaging System (Bio-Rad Laboratories, RRID: SCR_019037) and analyzed with Image LabTM software (Bio-Rad, RRID: SCR_014210). Samples were run at least two times and results from independent runs were averaged with a correction factor: Correction factor gel B = average of (OD protein of interest/OD β-actin for each sample loaded in gel A)/(OD protein of interest/OD β-actin for the same sample loaded in gel B) [36]. Example of full-size cropped immunoblots related to the protein expression levels measured in the whole homogenate, post-synaptic and cytosolic fractions of the striatum are presented in Figs. (**S1**-**S3**) and representative immunoblots for each protein are shown in Figs. (**1F**, **2D**, **3E**, **4D**).

### 2.3 Statistical Analysis

Data were collected in individual animals (independent determinations) and are shown as means ± standard errors. For each experiment, the normality of residuals was tested with the Kolmogorov-Smirnov test. Molecular changes in protein levels produced by genotype with normal distribution were analyzed by unpaired Student’s t-test (t), using as control condition DAT^+/+^ animals, in comparison to DAT^-/-^rats. Data with a non-normal distribution were analyzed by the Mann-Whitney test (U). Subjects were eliminated from the final dataset if their data deviated from the mean by 2 SDs. Prism 9 (GraphPad Software Prism v10, San Diego, CA, USA, RRID: SCR_002798) was used to analyze all data. Significance for all tests was assumed at *p <* 0.05.

## 3 RESULTS

To acquire an overall view of how the striatal GABAergic neurotransmission can be modulated by the increased dopaminergic tone that characterizes DAT^-/-^ rats, we first measured presynaptic GABAergic determinants. In the striatum homogenate of DAT^-/-^ rats, we found reduced expression of the vesicular transporter vGAT, which loads GABA in presynaptic vesicles [37], indicating decreased vesicle packaging and GABA release (Fig. **1A**: -22% *vs.* DAT^+/+^, t = 2.284, *p =* 0.0455). Such reduction was accompanied in the cytosolic fraction by a reduced expression of GAD67, one of the two main enzymes involved in GABA synthesis, predominately located in the neuron cell body (Fig. **1B**: -30% *vs.* DAT^+/+^, t = 2.605, *p =* 0.0314) and by an increased expression of GAD65 (Fig. **1C**: +56%, t = 3.129, *p =* 0.0128), enriched in the synaptic terminal and responsible for regulating vesicular GABA synthesis [38, 39]. The stability of the GABAergic synapse also relies on the reuptake of GABA, which is mainly carried out by the transporters GAT1 and GAT3, reduced in the homogenate of DAT^-/-^ rats (GAT1 Fig. **1D**: -16%, t = 4.743, *p =* 0.0011; GAT3 Fig. **1E**: -15%, t = 3.357, *p =* 0.0073).

We next measured the expression of calcium-binding proteins in the homogenate, which are important not only as markers for neuronal discrete subpopulations but also for the maintenance of proper GABA transmission [40, 41]. We found reduced expression of parvalbumin (Fig. **2A**: -38%, t = 3.958, *p =* 0.0042) and no change in the levels of calbindin (Fig. **2B**: +6%, t = 0.8808, *p =* 0.3991). Further, we detected a reduced expression of PGC1α (Fig. **2C**: -13%, U = 5, *p =* 0.0411), a protein critical for the maintenance of the proper functioning of parvalbumin-positive interneurons.

We next moved to the analysis of GABA-A receptor subunits and GABA-B receptor expression. In particular, we observed an overall reduction of the GABA-A receptor subunits α1 GABA-A (Fig. **3A**: -19%, t = 5.152, *p =* 0.0004), α2 GABA-A (Fig. **3B**: -14%, U = 5, *p =* 0.0411) and β1 GABA-A (Fig. **3C**: -16%, t = 3.052, *p =* 0.0122). The expression of the GABA-B R1 subunit was reduced as well (Fig. **3D**: -12%, U = 2, *p =* 0.0087).

We next analyzed the expression of NL-2, a postsynaptic adhesion molecule important for stabilizing GABA-A receptors in the postsynaptic density, and found its levels reduced (Fig. **4A**: -21%, t = 2.740, *p =* 0.0255). Since NL-2 is responsible for recruiting the main scaffolding protein of GABA receptors, *i.e*., gephyrin, we also measured its expression in the post-synaptic density and found it reduced as well (Fig. **4B**: -28%, t = 3.995, *p =* 0.0040).

Finally, we observed reduced expression of reelin in the homogenate (Fig. **4C**: -15%, t = 2.948, *p =* 0.0146), a glycoprotein that interferes with proper GABA homeostasis, by influencing the expression of GAD67 and the maturation of parvalbumin interneurons.

## 4 DISCUSSION

Our results reveal that the normal functioning of DAT is pivotal for the maintenance of striatal GABAergic homeostasis. In fact, under a condition of elevated dopaminergic tone, such as that observed in the striatum of DAT^-/-^ rats, the GABAergic homeostasis is indeed affected, as virtually all the GABAergic determinants herein examined are reduced. To sum up, we found a marked deficit in the expression of GABAergic proteins contributing to synthesis, storage, and reuptake as well as in the post-synaptic signal transduction, suggesting that, in a hyperdopaminergic state, the inhibitory GABAergic neurotransmission is toned down.

Changes in GABAergic molecular markers occur at both pre- and post-synaptic terminals. If we analyze the presynaptic compartment, we found a reduction of the vesicular transporter of GABA, vGAT, which suggests reduced packaging of the neurotransmitter into vesicles and reduced release of vesicular GABA. We also found reduced GAD67 expression, indicative of reduced non-vesicular GABA release [42]. The reduced ability to sequester GABA into vesicles may lead to a higher concentration of cytosolic GABA, and this may lay the foundation for the reduction in the expression of GAD67, which is mainly responsible for the synthesis of cytosolic GABA [38, 39]. However, the reduced storage of GABA in vesicles and the reduced vesicular release of GABA may be counteracted by the increased expression of GAD65, enriched in the nerve terminal and thought to regulate vesicular GABA synthesis, perhaps an attempt to maintain the physiological concentration of GABA at the synapse. The opposite effect on the expression of GAD65 and GAD67 may also suggest that these two proteins are regulated by different dopaminergic receptors, such as D1 or D2 receptors [43].

The homeostasis of the GABAergic synapse is critically dependent upon the reuptake of GABA, which is mainly accounted for by GAT1 and GAT3, which uptake the largest proportion of GABA in the central nervous system. GAT1 is strongly expressed in the striatum [44], whereas also GAT3 has been shown to have an active role [45]. We found a significant reduction of both GAT1 and GAT3, which may have multiple significance. In fact, the reduction can act as a buffer to the reduced release to maintain the physiological concentration of GABA in the synapse. However, it is widely established that membrane transporters not only terminate synaptic transmission through reuptake and regulate neurotransmitter spillover to neighboring synapses but are also responsible for the replenishment of the vesicles [46, 47]. This is also true for GABA [48], suggesting that reduced expression of GAT1 and GAT3 may indeed influence presynaptic GABA homeostasis by reducing the GABA availability for filling storage vesicles. Notably, the reduction of the main glial GABA transporter, *i.e*., GAT3, reveals that glial cells also play a role in counteracting the reduced release from the presynaptic terminal.

Another important observation is the marked reduction in the expression of parvalbumin. This clearly suggests a reduced inhibitory tone in the striatum. Interestingly, we also measured the expression of another calcium-binding protein, *i.e*., calbindin, which is unaffected by hyperdopaminergia, suggesting a selective loss of parvalbumin-expressing neurons: thus, the possibility exists that the reduced expression of parvalbumin, *i.e*., the calcium-binding protein with the higher calcium binding capacity, reduces the ability of the striatal cell of DAT^-/-^ rats to buffer calcium and may alter the excitatory/inhibitory balance. Of note, GAD67 and GAT1 colocalize in parvalbumin-expressing striatal neurons [44], suggesting that the reduction of parvalbumin observed herein may contribute to further alter such balance through the reduced GABA synthesis [49], which can be though compensated by a reduced GABA reuptake.

Another protein that colocalizes with GABAergic interneurons, including those positive for parvalbumin, is PGC1α [28, 50]. It has been proposed that PGC1α plays a unique role in gene programs specific for GABAergic interneurons [51]. PGC1α appears to be required for normal parvalbumin-positive interneuron function, and PGC1α colocalizes with GAD67 [28], suggesting that the herein observed reduction in the expression of PGC1α may influence parvalbumin and GAD67 expression and contribute to the overall tone down of the GABAergic neurotransmission. Notably, it is known that, when stimulated, extrasynaptic glutamate receptors may impair CREB-PGC1-mediated signaling [52]: interestingly, we have recently demonstrated that an elevated dopaminergic tone leads to increased lateral movement of NMDA receptors, which may lay the foundation for the herein reduction of PGC1α expression [5]. Thus, in a global picture, under a condition of elevated dopaminergic tone, we have insufficient GABA synthesis, a reduced GABA release and a less efficient recycle of released GABA.

Our findings also show a general reduction of GABA-A and GABA-B receptor expression. Evidence exists showing that α1 and α2 subunits are regulated by dopamine at the level of medium-spiny neurons [53]. Accordingly, as a consequence of the elevated dopaminergic tone in the striatum of DAT^-/-^ rats, the post-synaptic GABAergic response might be weakened, as shown by the reduced expression of the main GABA-A receptor subunits and of GABA B receptors. An effect that may also influence the functional properties of these receptors. In fact, the alteration in GABA-A receptor subunit composition may lead to anomalous GABA-A currents caused by the reduced expression of the α1 subunit that regulates fast inhibitory synaptic currents [54]. We also observed a significant reduction of the α2 subunit, which is highly expressed in striatal medium spiny neurons. This evidence may be functionally relevant as the α2 subunit is reduced in the striatum of an animal model of Huntington’s disease, consistent with the decreased GABAergic current in the medium spiny neurons of this animal model [55]. The reduction in GABA-B receptor expression aligns with what is observed under repeated exposure to cocaine, a condition similar to that in dopamine transporter knockout animals. [56]. Such downregulation may compromise the ability of these receptors to inhibit the dopaminergic tone, and it may partially contribute to explaining the dopamine elevation at the extracellular level.

The set of hyperdopaminergia-induced postsynaptic GABAergic changes involves the reduced expression of NL-2, suggesting reduced synaptic strength in GABAergic synapses and reduced recruitment of the postsynaptic scaffold protein gephyrin, known to stabilize the GABA-A receptor in the postsynaptic density [25]. Notably, the reduction of gephyrin may suggest an increased lateral movement of GABA-A receptors [57, 58]. These lines of evidence may contribute to explaining the reduced synaptic expression of GABA-A receptors in a situation of hyperdopaminergia. In line with the global picture mentioned above, less GABA released at the extracellular level would bind a lower number of less stable GABA receptors, leading to a further reduced inhibitory drive.

Such depressed GABAergic tone is further denoted by the reduced expression of reelin. Reelin is an extracellular glycoprotein preferentially synthesized by GABAergic neurons in the adult rodent brain [59]. The critical role of this protein in the GABAergic system is strengthened by the evidence that reelin influences the expression of GAD67 [60] as well as of parvalbumin interneurons [61, 62]. In addition, the reduction of reelin may contribute to explain, at least partially, the reduction in dendritic spine density we have observed in the striatum of DAT^-/-^ rats [5] since evidence exists that reelin regulates dendritic spine density as well as the reduction of GAD67 expression [63].

## CONCLUSION

In conclusion, our data indicate a multifaceted down-regulation of striatal GABAergic transmission in DAT^-/-^ rats, suggesting an alteration of the optimal tuning of GABAergic synapses under a condition of elevated dopaminergic neurotransmission. Whether it reflects the general loss of GABAergic MSNs due to the overactivation of D1 dopamine receptors, as observed in DAT^-/-^ mice [11], deserves further investigation.

We are aware that this paper holds some limitations. First, we do not have electrophysiological data to sustain the potential functional relevance of our data and, therefore, we primarily rely on protein measures to infer changes in function; we are reasonably sure of our assumption as we have shown a coordinated series of changes in GABAergic determinants, which converge on a cohesive picture indicative of a reduced GABAergic tone. Second, we cannot rule out the possibility that such changes in striatal GABAergic neurotransmission may influence other brain regions, as they may be involved in tuning up the cortical neurotransmission causing hyperexcitability [12]. Last, we cannot generalize our findings to both sexes since in this study we focused only on male rats.

The changes herein illustrated may play a role in different disease conditions. For instance, it has been demonstrated how reduced GABA function contributes to schizophrenia and autism [64-67]. Interestingly, reduced expression of parvalbumin [68], GAD67, and reelin [69] are highly replicated findings in the brains of schizophrenia patients. Further, impaired striatal GABAergic neurotransmission has been consistently observed across different animal models of Huntington’s disease and from autoptic brain samples from Huntington’s disease patients [70-72]. In addition, impaired GABAergic functions have been found in other hyperkinetic movement disorders such as dystonia [73, 74] and Tourette syndrome [75], the latter showing reduced parvalbumin-expressing interneurons in the striatum [76].

## Data Availability

The data that support the findings of this study are available from the corresponding author upon reasonable request.

## LIST OF ABBREVIATIONS

DAT: Dopamine Transporter
GABA: γ-Aminobutyric acid
GAT(1/3): GABA Transporters (1/3)
GAD(67/65): Glutamate Decarboxylase (67/65)
NL-2: Neuroligin-2
vGAT: Vesicular GABA Transporter

## References

[1] Leo D., Sukhanov I., Zoratto F., Illiano P., Caffino L., Sanna F., Messa G., Emanuele M., Esposito A., Dorofeikova M., Budygin E.A., Mus L., Efimova E.V., Niello M., Espinoza S., Sotnikova T.D., Hoener M.C., Laviola G., Fumagalli F., Adriani W., Gainetdinov R.R. (2018). Pronounced hyperactivity, cognitive dysfunctions, and BDNF dysregulation in dopamine transporter knock-out rats.. J. Neurosci..

[2] Giros B., Jaber M., Jones S.R., Wightman R.M., Caron M.G. (1996). Hyperlocomotion and indifference to cocaine and amphetamine in mice lacking the dopamine transporter.. Nature.

[3] Sora I., Wichems C., Takahashi N., Li X.F., Zeng Z., Revay R., Lesch K.P., Murphy D.L., Uhl G.R. (1998). Cocaine reward models: Conditioned place preference can be established in dopamine- and in serotonin-transporter knockout mice.. Proc. Natl. Acad. Sci. USA.

[4] Targa G., Mottarlini F., Rizzi B., Leo D., Caffino L., Fumagalli F. (2023). Dysregulation of AMPA receptor trafficking and intracellular vesicular sorting in the prefrontal cortex of dopamine transporter knock-out rats.. Biomolecules.

[5] Caffino L., Targa G., Mottarlini F., Thielens S., Rizzi B., Villers A. (2024). Memantine-induced functional rewiring of the glutamate synapse in the striatum of dopamine transporter knockout rats.. Br. J. Pharmacol..

[6] Kubota Y., Inagaki S., Kito S., Wu J.Y. (1987). Dopaminergic axons directly make synapses with GABAergic neurons in the rat neostriatum.. Brain Res..

[7] Pickel V.M., Towle A.C., Joh T.H., Chan J. (1988). Gamma‐aminobutyric acid in the medial rat nucleus accumbens: Ultrastructural localization in neurons receiving monosynaptic input from catecholaminergic afferents.. J. Comp. Neurol..

[8] Flores-Hernandez J., Hernandez S., Snyder G.L., Yan Z., Fienberg A.A., Moss S.J., Greengard P., Surmeier D.J.D. (2000). (1) dopamine receptor activation reduces GABA(A) receptor currents in neostriatal neurons through a PKA/DARPP-32/PP1 signaling cascade.. J. Neurophysiol..

[9] Morello F., Partanen J. (2015). Diversity and development of local inhibitory and excitatory neurons associated with dopaminergic nuclei.. FEBS Lett..

[10] Tepper J.M., Martin L.P., Anderson D.R. (1995). GABAA receptor-mediated inhibition of rat substantia nigra dopaminergic neurons by pars reticulata projection neurons.. J. Neurosci..

[11] Cyr M., Beaulieu J.M., Laakso A., Sotnikova T.D., Yao W.D., Bohn L.M., Gainetdinov R.R., Caron M.G. (2003). Sustained elevation of extracellular dopamine causes motor dysfunction and selective degeneration of striatal GABAergic neurons.. Proc. Natl. Acad. Sci. USA.

[12] Reinwald J.R., Gass N., Mallien A.S., Sartorius A., Becker R., Sack M., Falfan-Melgoza C., Clemm von Hohenberg C., Leo D., Pfeiffer N., Middelman A., Meyer-Lindenberg A., Homberg J.R., Weber-Fahr W., Gass P. (2022). Dopamine transporter silencing in the rat: Systems-level alterations in striato-cerebellar and prefrontal-midbrain circuits.. Mol. Psychiatry.

[13] Boccalaro I.L., Cristiá-Lara L., Schwerdel C., Fritschy J.M., Rubi L. (2019). Cell type‐specific distribution of GABAA receptor subtypes in the mouse dorsal striatum.. J. Comp. Neurol..

[14] Koós T., Tepper J.M. (1999). Inhibitory control of neostriatal projection neurons by GABAergic interneurons.. Nat. Neurosci..

[15] Centonze D., Grande C., Saulle E., Martín A.B., Gubellini P., Pavón N., Pisani A., Bernardi G., Moratalla R., Calabresi P. (2003). Distinct roles of D1 and D5 dopamine receptors in motor activity and striatal synaptic plasticity.. J. Neurosci..

[16] Wiltschko A.B., Pettibone J.R., Berke J.D. (2010). Opposite effects of stimulant and antipsychotic drugs on striatal fast-spiking interneurons.. Neuropsychopharmacology.

[17] Bracci E., Centonze D., Bernardi G., Calabresi P. (2002). Dopamine excites fast-spiking interneurons in the striatum.. J. Neurophysiol..

[18] Tritsch N.X., Ding J.B., Sabatini B.L. (2012). Dopaminergic neurons inhibit striatal output through non-canonical release of GABA.. Nature.

[19] Möhler H. (2006). GABAA receptor diversity and pharmacology.. Cell Tissue Res..

[20] Macdonald R.L., Olsen R.W. (1994). GABAA receptor channels.. Annu. Rev. Neurosci..

[21] Kim J.J., Hibbs R.E. (2021). Direct structural insights into GABAA receptor pharmacology.. Trends Biochem. Sci..

[22] Luscher B., Fuchs T., Kilpatrick C.L. (2011). GABAA receptor trafficking-mediated plasticity of inhibitory synapses.. Neuron.

[23] Nusser Z., Cull-Candy S., Farrant M. (1997). Differences in synaptic GABA(A) receptor number underlie variation in GABA mini amplitude.. Neuron.

[24] Otis T.S., De Koninck Y., Mody I. (1994). Lasting potentiation of inhibition is associated with an increased number of gamma-aminobutyric acid type A receptors activated during miniature inhibitory postsynaptic currents.. Proc. Natl. Acad. Sci. USA.

[25] Poulopoulos A., Aramuni G., Meyer G., Soykan T., Hoon M., Papadopoulos T., Zhang M., Paarmann I., Fuchs C., Harvey K., Jedlicka P., Schwarzacher S.W., Betz H., Harvey R.J., Brose N., Zhang W., Varoqueaux F. (2009). Neuroligin 2 drives postsynaptic assembly at perisomatic inhibitory synapses through gephyrin and collybistin.. Neuron.

[26] Varoqueaux F., Jamain S., Brose N. (2004). Neuroligin 2 is exclusively localized to inhibitory synapses.. Eur. J. Cell Biol..

[27] Patrizi A., Scelfo B., Viltono L., Briatore F., Fukaya M., Watanabe M., Strata P., Varoqueaux F., Brose N., Fritschy J.M., Sassoè-Pognetto M. (2008). Synapse formation and clustering of neuroligin-2 in the absence of GABAA receptors.. Proc. Natl. Acad. Sci. USA.

[28] Cowell R.M., Blake K.R., Russell J.W. (2007). Localization of the transcriptional coactivator PGC‐1α to GABAergic neurons during maturation of the rat brain.. J. Comp. Neurol..

[29] Alcántara S., Ruiz M., D’Arcangelo G., Ezan F., de Lecea L., Curran T., Sotelo C., Soriano E. (1998). Regional and cellular patterns of reelin mRNA expression in the forebrain of the developing and adult mouse.. J. Neurosci..

[30] Herz J., Chen Y. (2006). Reelin, lipoprotein receptors and synaptic plasticity.. Nat. Rev. Neurosci..

[31] Carbery I.D., Ji D., Harrington A., Brown V., Weinstein E.J., Liaw L., Cui X. (2010). Targeted genome modification in mice using zinc-finger nucleases.. Genetics.

[32] Geurts A.M., Cost G.J., Freyvert Y., Zeitler B., Miller J.C., Choi V.M. (2009). Knockout rats produced using designed zinc finger nucleases.. Science.

[33] Mottarlini F., Targa G., Bottan G., Tarenzi B., Fumagalli F., Caffino L. (2022). Cortical reorganization of the glutamate synapse in the activity‐based anorexia rat model: Impact on cognition.. J. Neurochem..

[34] Piva A., Caffino L., Mottarlini F., Pintori N., Castillo Díaz F., Fumagalli F., Chiamulera C. (2021). Metaplastic effects of ketamine and MK-801 on glutamate receptors expression in rat medial prefrontal cortex and hippocampus.. Mol. Neurobiol..

[35] Caffino L., Targa G., Mallien A.S., Mottarlini F., Rizzi B., Homberg J.R. (2024). Chronic lithium treatment alters NMDA and AMPA receptor synaptic availability and dendritic spine organization in the rat hippocampus.. Curr. Neuropharmacol..

[36] Caffino L., Verheij M.M.M., Roversi K., Targa G., Mottarlini F., Popik P., Nikiforuk A., Golebiowska J., Fumagalli F., Homberg J.R. (2020). Hypersensitivity to amphetamine’s psychomotor and reinforcing effects in serotonin transporter knockout rats: Glutamate in the nucleus accumbens.. Br. J. Pharmacol..

[37] Dumoulin A., Rostaing P., Bedet C., Lévi S., Isambert M.F., Henry J.P., Triller A., Gasnier B. (1999). Presence of the vesicular inhibitory amino acid transporter in GABAergic and glycinergic synaptic terminal boutons.. J. Cell Sci..

[38] Sheikh S.N., Martin S.B., Martin D.L. (1999). Regional distribution and relative amounts of glutamate decarboxylase isoforms in rat and mouse brain.. Neurochem. Int..

[39] Soghomonian J.J., Martin D.L. (1998). Two isoforms of glutamate decarboxylase: Why?. Trends Pharmacol. Sci..

[40] Baimbridge K.G., Celio M.R., Rogers J.H. (1992). Calcium-binding proteins in the nervous system.. Trends Neurosci..

[41] Ferguson B.R., Gao W.J. (2018). PV interneurons: Critical regulators of E/I balance for prefrontal cortex-dependent behavior and psychiatric disorders.. Front. Neural Circuits.

[42] Esclapez M., Tillakaratne N.J., Kaufman D.L., Tobin A.J., Houser C.R. (1994). Comparative localization of two forms of glutamic acid decarboxylase and their mRNAs in rat brain supports the concept of functional differences between the forms.. J. Neurosci..

[43] Laprade N., Soghomonian J.J. (1995). Differential regulation of mRNA levels encoding for the two isoforms of glutamate decarboxylase (GAD65 and GAD67) by dopamine receptors in the rat striatum.. Brain Res. Mol. Brain Res..

[44] Augood S.J., Herbison A.E., Emson P.C. (1995). Localization of GAT-1 GABA transporter mRNA in rat striatum: Cellular coexpression with GAD67 mRNA, GAD67 immunoreactivity, and parvalbumin mRNA.. J. Neurosci..

[45] Kirmse K., Kirischuk S., Grantyn R. (2009). Role of GABA transporter 3 in GABAergic synaptic transmission at striatal output neurons.. Synapse.

[46] Mandela P., Ordway G.A. (2006). The norepinephrine transporter and its regulation.. J. Neurochem..

[47] Nepal B., Das S., Reith M.E., Kortagere S. (2023). Overview of the structure and function of the dopamine transporter and its protein interactions.. Front. Physiol..

[48] Conti F., Melone M., Fattorini G., Bragina L., Ciappelloni S. (2011). A role for GAT-1 in presynaptic GABA homeostasis?. Front. Cell. Neurosci..

[49] Zeng C., Lu Y., Wei X., Sun L., Wei L., Ou S., Huang Q., Wu Y. (2024). Parvalbumin regulates GAD expression through calcium ion concentration to affect the balance of Glu-GABA and improve KA-induced status epilepticus in PV-Cre transgenic mice.. ACS Chem. Neurosci..

[50] Lucas E.K., Markwardt S.J., Gupta S., Meador-Woodruff J.H., Lin J.D., Overstreet-Wadiche L., Cowell R.M. (2010). Parvalbumin deficiency and GABAergic dysfunction in mice lacking PGC-1α.. J. Neurosci..

[51] Wang J., Song H.R., Guo M.N., Ma S.F., Yun Q., Zhang W.N. (2020). Adult conditional knockout of PGC-1α in GABAergic neurons causes exaggerated startle reactivity, impaired short-term habituation and hyperactivity.. Brain Res. Bull..

[52] Okamoto S., Pouladi M.A., Talantova M., Yao D., Xia P., Ehrnhoefer D.E., Zaidi R., Clemente A., Kaul M., Graham R.K., Zhang D., Vincent Chen H-S., Tong G., Hayden M.R., Lipton S.A. (2009). Balance between synaptic versus extrasynaptic NMDA receptor activity influences inclusions and neurotoxicity of mutant huntingtin.. Nat. Med..

[53] Goffin D., Ali A.B., Rampersaud N., Harkavyi A., Fuchs C., Whitton P.S., Nairn A.C., Jovanovic J.N. (2010). Dopamine-dependent tuning of striatal inhibitory synaptogenesis.. J. Neurosci..

[54] Vicini S., Ferguson C., Prybylowski K., Kralic J., Morrow A.L., Homanics G.E. (2001). GABA(A) receptor alpha1 subunit deletion prevents developmental changes of inhibitory synaptic currents in cerebellar neurons.. J. Neurosci..

[55] Du X., Hao H., Yang Y., Huang S., Wang C., Gigout S., Ramli R., Li X., Jaworska E., Edwards I., Deuchars J., Yanagawa Y., Qi J., Guan B., Jaffe D.B., Zhang H., Gamper N. (2017). Local GABAergic signaling within sensory ganglia controls peripheral nociceptive transmission.. J. Clin. Invest..

[56] Frankowska M., Wydra K., Faron-Górecka A., Zaniewska M., Kuśmider M., Dziedzicka-Wasylewska M., Filip M. (2008). Neuroadaptive changes in the rat brain GABAB receptors after withdrawal from cocaine self-administration.. Eur. J. Pharmacol..

[57] Essrich C., Lorez M., Benson J.A., Fritschy J.M., Lüscher B. (1998). Postsynaptic clustering of major GABAA receptor subtypes requires the γ2 subunit and gephyrin.. Nat. Neurosci..

[58] Jacob T.C., Bogdanov Y.D., Magnus C., Saliba R.S., Kittler J.T., Haydon P.G., Moss S.J. (2005). Gephyrin regulates the cell surface dynamics of synaptic GABAA receptors.. J. Neurosci..

[59] Pesold C., Impagnatiello F., Pisu M.G., Uzunov D.P., Costa E., Guidotti A., Caruncho H.J. (1998). Reelin is preferentially expressed in neurons synthesizing γ-aminobutyric acid in cortex and hippocampus of adult rats.. Proc. Natl. Acad. Sci. USA.

[60] Romano E., Fuso A., Laviola G. (2013). Nicotine restores Wt-like levels of reelin and GAD67 gene expression in brain of heterozygous reeler mice.. Neurotox. Res..

[61] Marrone M.C., Marinelli S., Biamonte F., Keller F., Sgobio C.A., Ammassari-Teule M., Bernardi G., Mercuri N.B. (2006). Altered cortico‐striatal synaptic plasticity and related behavioural impairments in reeler mice.. Eur. J. Neurosci..

[62] Pardo M., Gregorio S., Montalban E., Pujadas L., Elias-Tersa A., Masachs N., Vílchez-Acosta A., Parent A., Auladell C., Girault J.A., Vila M., Nairn A.C., Manso Y., Soriano E. (2023). Adult-specific Reelin expression alters striatal neuronal organization: implications for neuropsychiatric disorders.. Front. Cell. Neurosci..

[63] Liu W.S., Pesold C., Rodriguez M.A., Carboni G., Auta J., Lacor P., Larson J., Condie B.G., Guidotti A., Costa E. (2001). Down-regulation of dendritic spine and glutamic acid decarboxylase 67 expressions in the reelin haploinsufficient heterozygous reeler mouse.. Proc. Natl. Acad. Sci. USA.

[64] Gonzalez-Burgos G., Hashimoto T., Lewis D.A. (2010). Alterations of cortical GABA neurons and network oscillations in schizophrenia.. Curr. Psychiatry Rep..

[65] Lisman J.E., Coyle J.T., Green R.W., Javitt D.C., Benes F.M., Heckers S., Grace A.A. (2008). Circuit-based framework for understanding neurotransmitter and risk gene interactions in schizophrenia.. Trends Neurosci..

[66] Williams S., Boksa P. (2010). Gamma oscillations and schizophrenia.. J. Psychiatry Neurosci..

[67] Wilson T.W., Rojas D.C., Reite M.L., Teale P.D., Rogers S.J. (2007). Children and adolescents with autism exhibit reduced MEG steady-state gamma responses.. Biol. Psychiatry.

[68] Marín O. (2024). Parvalbumin interneuron deficits in schizophrenia.. Eur. Neuropsychopharmacol..

[69] Guidotti A., Auta J., Davis J.M., Gerevini V.D.G., Dwivedi Y., Grayson D.R., Impagnatiello F., Pandey G., Pesold C., Sharma R., Uzunov D., Costa E. (2000). Decrease in reelin and glutamic acid decarboxylase67 (GAD67) expression in schizophrenia and bipolar disorder: A postmortem brain study.. Arch. Gen. Psychiatry.

[70] Du Z., Tertrais M., Courtand G., Leste-Lasserre T., Cardoit L., Masmejean F., Halgand C., Cho Y.H., Garret M. (2017). Differential alteration in expression of striatal GABAAR subunits in mouse models of huntington’s disease.. Front. Mol. Neurosci..

[71] Hsu Y.T., Chang Y.G., Chern Y. (2018). Insights into GABAA ergic system alteration in Huntington’s disease.. Open Biol..

[72] Reiner A., Shelby E., Wang H., DeMarch Z., Deng Y., Guley N.H., Hogg V., Roxburgh R., Tippett L.J., Waldvogel H.J., Faull R.L.M. (2013). Striatal parvalbuminergic neurons are lost in Huntington’s disease: Implications for dystonia.. Mov. Disord..

[73] Gittis A.H., Leventhal D.K., Fensterheim B.A., Pettibone J.R., Berke J.D., Kreitzer A.C. (2011). Selective inhibition of striatal fast-spiking interneurons causes dyskinesias.. J. Neurosci..

[74] Levy L.M., Hallett M. (2002). Impaired brain GABA in focal dystonia.. Ann. Neurol..

[75] Kalanithi P.S.A., Zheng W., Kataoka Y., DiFiglia M., Grantz H., Saper C.B., Schwartz M.L., Leckman J.F., Vaccarino F.M. (2005). Altered parvalbumin-positive neuron distribution in basal ganglia of individuals with Tourette syndrome.. Proc. Natl. Acad. Sci. USA.

[76] Kataoka Y., Kalanithi P.S.A., Grantz H., Schwartz M.L., Saper C., Leckman J.F., Vaccarino F.M. (2010). Decreased number of parvalbumin and cholinergic interneurons in the striatum of individuals with Tourette syndrome.. J. Comp. Neurol..

