# Supplementary material for: Altered GABAergic Homeostasis in the Striatum of Dopamine Transporter Knockout Rats
Source: Curr Neuropharmacol. 2025 Apr 18;23(11):1470–80. doi: 10.2174/011570159X370747250404060428 (PMC12613098; doi:10.2174/011570159X370747250404060428)

Supplementary Material

Altered GABAergic Homeostasis in the Striatum of Dopamine Transporter Knockout Rats

Giorgia Targa[1,#], Beatrice Rizzi[1,2,#], Francesca Mottarlini[1], Raul R. Gainetdinov[3,4], Damiana Leo[5], Fabio Fumagalli[1,*] and Lucia Caffino[1]

[1]Department of Pharmacological and Biomolecular Sciences 'Rodolfo Paoletti', Università degli Studi di Milano, Via Balzaretti 9, 20133, Milan, Italy; [2]Center for Neuroscience, University of Camerino, Camerino, Italy; [3]Institute of Translational Biomedicine, St. Petersburg State University, 7/9 Universitetskaya Emb., 199034 St. Petersburg, Russia; [4]St. Petersburg University Hospital, St. Petersburg State University, Fontanka River Emb. 154, 190121 St. Petersburg, Russia; [5]Department of Neurosciences, University of Mons, 6 Avenue du Champ de Mars, 7000, Mons, Belgium

1875-6190/25 $65.00+.00 **© 2025 Bentham Science Publishers**

Supplementary figure 1. Example of full-size cropped immunoblot related to the expression levels of NL-2, Gephyrin and β-actin measured in the Post-Synaptic density (A, B) and of GAD67, GAD65 and β-actin measured in the cytosolic fraction (C, D) of Striatum of DAT $^{+/+}$ and DAT $^{-/-}$ rats presented in **figures 1**, **2** and **4**.

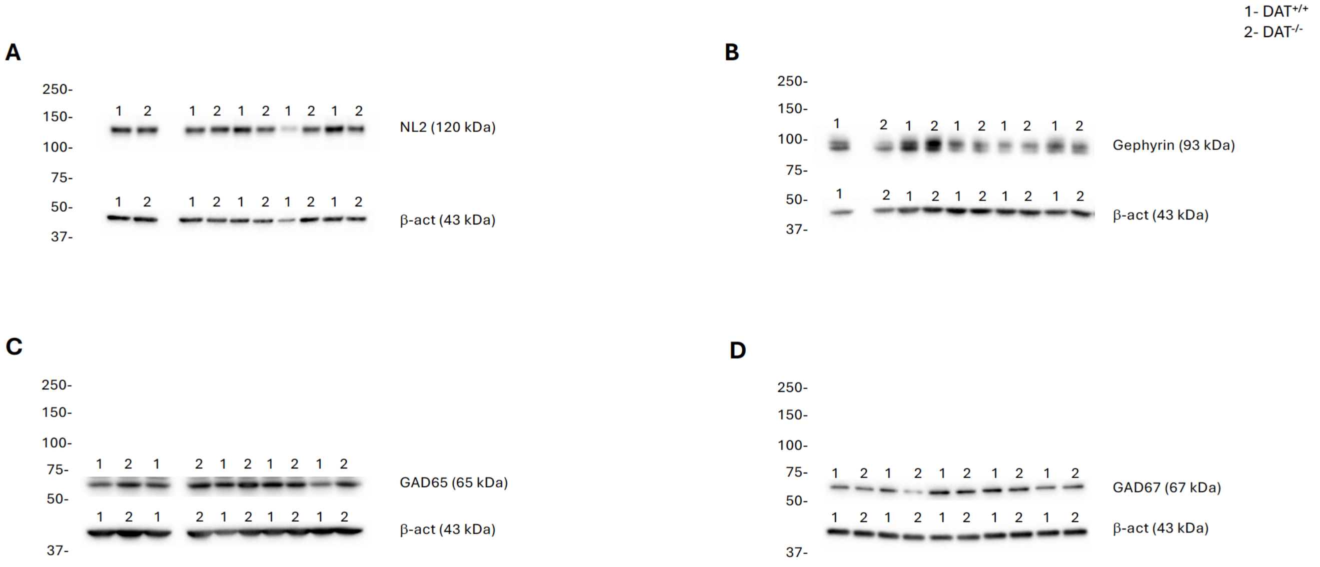

Supplementary figure 2. Example of full-size cropped immunoblot related to the expression levels of Reelin, GABA-B R1, PCG1α, GAT1, GAT3, vGAT, α1 GABA-A, α2 GABA-A, β1 GABA-A, β-actin, Calbindin (A) and Parvalbumin (B) measured in the homogenate of Striatum of DAT $^{+/+}$ and DAT $^{-/-}$ rats presented in **figures 1**, **2**, **3** and **4**.

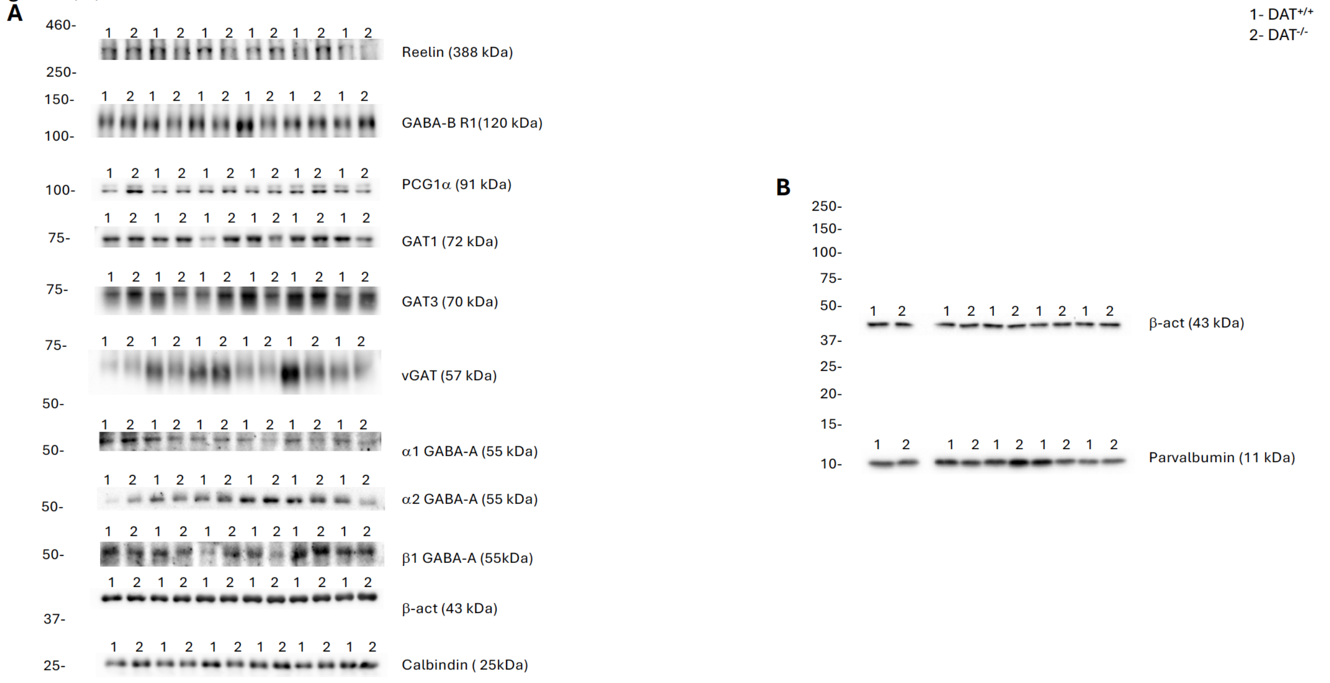

Supplementary figure 3. Merge of immunoblot signal used for quantification with scan of the cropped part of the membrane on which the respective primary antibody was incubated showing the prestained protein marker (A: HiMark Pre-Stained Protein Standard TermoFisher Scientific #LC5699; B: prestained protein marker Biorad #161-0373).

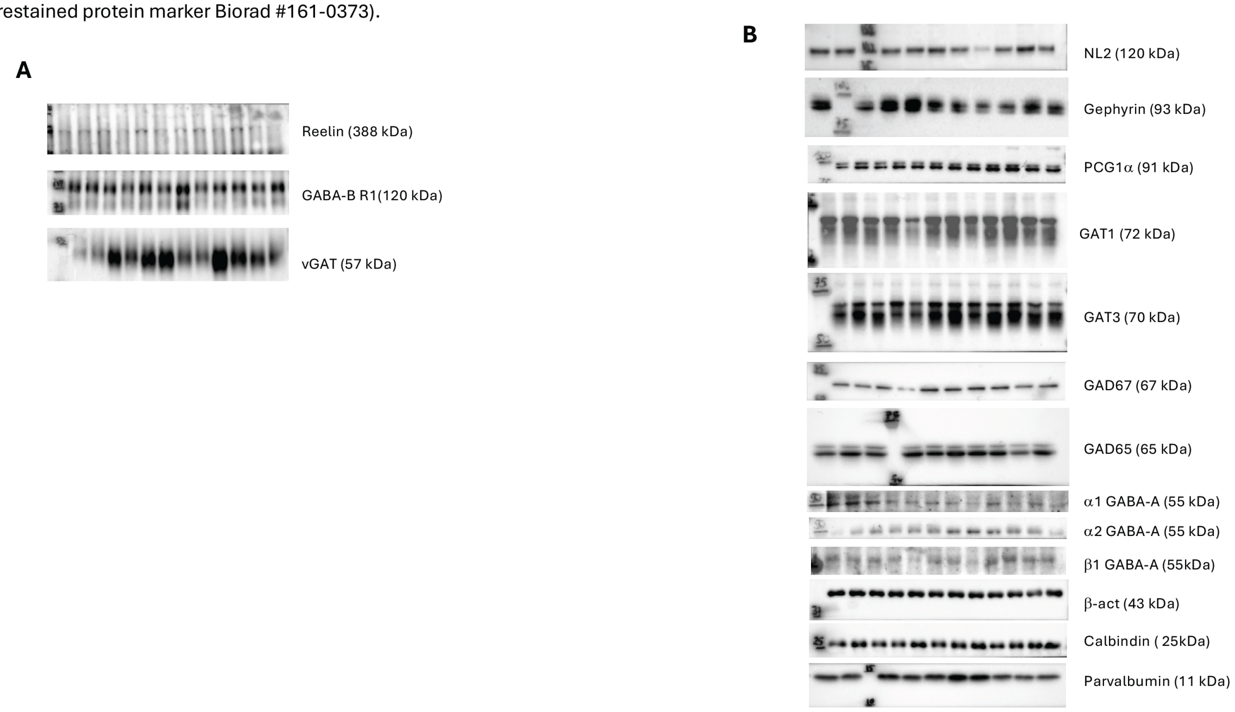

Supplement: Supplementary file 1 [file CN-23-11-1470_SD1.pdf]
